# Highly Crystalline K‐Intercalated Polymeric Carbon Nitride for Visible‐Light Photocatalytic Alkenes and Alkynes Deuterations

**DOI:** 10.1002/advs.201801403

**Published:** 2018-11-08

**Authors:** Chuntian Qiu, Yangsen Xu, Xin Fan, Dong Xu, Rika Tandiana, Xiang Ling, Yanan Jiang, Cuibo Liu, Lei Yu, Wei Chen, Chenliang Su

**Affiliations:** ^1^ SZU‐NUS Collaborative Center and International Collaborative Laboratory of 2D Materials for Optoelectronic Science and Technology of Ministry of Education College of Optoelectronic Engineering Shenzhen University Shenzhen 518060 China; ^2^ Engineering Technology Research Center for 2D Material Information Function Devices and Systems of Guangdong Province Shenzhen University Shenzhen 518060 China; ^3^ School of Chemistry and Chemical Engineering Yangzhou University Yangzhou Jiangsu 225002 China; ^4^ Department of Civil and Environmental Engineering National University of Singapore 1 Engineering Drive 2 117576 Singapore; ^5^ Department of Chemistry National University of Singapore 3 Science Drive 3 117543 Singapore

**Keywords:** alkene deuterations, crystalline KPCN, deuterated chemicals, hydrogen evolution, photocatalysis

## Abstract

In addition to the significance of photocatalytic hydrogen evolution, the utilization of the in situ generated H/D (deuterium) active species from water splitting for artificial photosynthesis of high value‐added chemicals is very attractive and promising. Herein, photocatalytic water splitting technology is utilized to generate D‐active species (i.e., D_ad_) that can be stabilized on anchored 2nd metal catalyst and are readily for tandem controllable deuterations of carbon–carbon multibonds to produce high value‐added D‐labeled chemicals/pharmaceuticals. A highly crystalline K cations intercalated polymeric carbon nitride (KPCN), rationally designed, and fabricated by a solid‐template induced growth, is served as an ultraefficient photocatalyst, which shows a greater than 18‐fold enhancement in the photocatalytic hydrogen evolution over the bulk PCN. The photocatalytic in situ generated D‐species by superior KPCN are utilized for selective deuteration of a variety of alkenes and alkynes by anchored 2nd catalyst, Pd nanoparticles, to produce the corresponding D‐labeled chemicals and pharmaceuticals with high yields and D‐incorporation. This work highlights the great potential of developing photocatalytic water splitting technology for artificial photosynthesis of value‐added chemicals instead of H_2_ evolution.

## 1 Introduction

Deuterium‐labeled compounds had found their significant utility in synthetic mechanistic study, mass spectrometry‐based quantification and pharmaceutical industry over the past few years.^1^ The first approval of deuterated drug deutetrabenazine (SD‐809) by the US Food and Drug Administration in 2017 represented a new milestone in heavy drugs/chemicals, which triggers intense emotions in the development of useful heavy drugs as well as new‐generation deuteration strategies.^2^ Selective installation of deuterium atoms in the target organic/pharmaceutical compounds is therefore of high synthetic interests but still remains challenge. The state of art C–H/C–D exchange process is a powerful deuteration strategy but often suffers from harsh reaction conditions, limited scope (mainly for deuteration of sp^2^ or sp bonds), or nonselective multiposition labeling.^3^ Controllable deuteration of organic functional groups (halides, carbonyls, alkenes, etc.) provides a promising route for selective installation of deuterium atoms in the target organic compounds, which therefore have attracted growing attention.^4^ C=C or/and C≡C bonds are ubiquitous in many important fine chemicals and natural products. However, there are surprisingly few reports for selective deuteration of C=C or/and C≡C bonds with green deuterium source to uniquely install deuteriums in unactived sp^3^ bonds. One example developed by Stokes' group uses expensive additives such as B_2_(OH)_4_ to engage and activate D_2_O for deuteriation of alkenes, where very limited deuteration examples were presented.^5^ Thus, developing a general approach combining new catalytic system with green and inexpensive deuterium source (D‐source) for selective deuteration of alkenes and alkynes with broad reaction scope is highly desired.

Photocatalytic water splitting over semiconductors for hydrogen evolution has become one of the most promising ways to convert solar‐energy into storage hydrogen energy.^6^ Replacing H_2_O by D_2_O, the photocatalytic water splitting technology is also a potential route to generate active D‐species (adsorbed D, D_ad_) from the cleavage of an O—D bond in a D_2_O molecule excited by light. The utilization of the in situ generated D‐source from heavy water splitting in the artificial photosynthesis of value‐added D‐labeled chemicals is very attractive but rarely investigated. In this regard, we envision a synergistic photocatalytic strategy to in situ generate active D‐sources stabilized by 2nd metal catalysts on semiconductors and utilized for sequentially controllable deuteration of alkenes and alkynes for valuable heavy chemicals/pharmaceuticals production. To achieve this scheme, one major challenge is to explore a highly efficient visible‐light‐response photocatalyst that is environment‐friendly, earth‐abundant, and low‐cost.

Polymeric carbon nitride (PCN), a metal‐free semiconductor has been identified as one of the most promising candidates, due to its easily preparation, environment‐benign, low toxic, and appropriated band gap for visible light‐driven photocatalysis.^7^ In addition, the inbuilt nitrogen functionalities (—NH_2_ and —NH—) and π‐bonded planar layered configurations endow PCN with a good platform to anchor desired metals (Ag, Pd or Pt) to form a multiple functional catalyst.^8^ Despite these advantages, low product yield, moderate photocatalytic activity, and especially the poor crystallinity of PCN greatly hamper its widespread applications. The traditional direct thermal polymerization method usually leads to low crystalline PCN due to the predominantly kinetic hindrance.^9^ Previous researches demonstrated that the photocatalytic activity of PCN is highly dependent on its electronic and crystalline structures.^10^ A well‐defined structure with an ordered molecular arrangement and a controllable morphology not only enhances electron and mass transfer but also facilitates the separation of electron‐hole pairs to promote redox reactions.^11^ Therefore, how to design and construct a well‐defined and highly condensed crystalline PCN to improve its photocatalytic efficiency is significative but challenging.

Herein, we have succeeded in the synthesis of a highly crystallized K cation intercalated polymeric carbon nitride (KPCN) by thermal condensation of melamine with KBr. The obtained KPCN showed a greater than 18‐fold enhancement for photocatalytic H_2_ evolution over the bulk PCN. The highly crystalline structure not only enhances electron and mass transfer but also facilitates the separation of electron‐hole pairs to promote redox reactions. When decorated with nano‐Pd, this synergistic catalytic composite can be utilized for selective deuteration of alkenes and alkynes with the in situ generated D‐species from heavy water splitting. This new alkene‐deuteration strategy is performed at room temperature, under ambient pressure, producing corresponding deuterated chemicals as well as pharmaceutical‐related compounds in good to excellent yields with high D‐incorporation.

## 2 Results and Discussion

**Figure 1:**
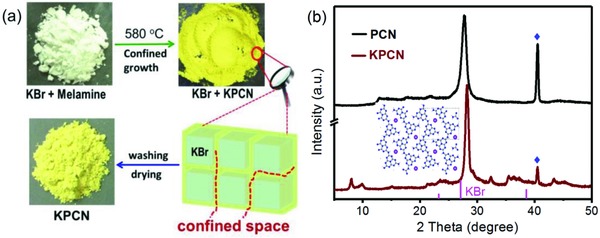
a) Schematic illustration of the KBr‐confinement growth of KPCN. b) XRD patterns of original PCN and KPCN. ♦ represent the Bragg peak of the Mo (JCPDS 42–1120) which acts as the reference to correct the intrinsic diffraction peak.

As illustrated in **Figure** **1**a, a newly formed KPCN was controllably synthesized via salt confinement growth followed by pyrolysis. Solid KBr was utilized as a solid template for the growth of KPCN because of its higher melting point (*T*_m_ = 730 °C) than the reaction temperature (580 °C). A high molar ratio of KBr/melamine (more than 4:3) provided confined spaces (the interspaces of KBr crystals), thus leading to the confined growth of KPCN within the KBr crystals (red dashed lines in Figure 1a) to give a crystalline structure. Additionally, the KBr salt could also act as a reactant which provides the K cations (about 9.73 wt%, inductively coupled plasma (ICP) analysis) inserted into the melon chains (the proposed structure inserted in Figure 1b) or layers for the formed joint melting phase between melamine and KBr.

To study the variation in the crystalline structure of KPCN, XRD characterization was performed (Figure 1b). Obviously, KPCN displayed a different XRD pattern and showed one main peak that was red‐shifted by ≈0.5° to 28.17° in comparison to PCN (27.68°), corresponding to an interlayer *d*‐spacing of 0.316 nm. This interlayer spacing is smaller than that of PCN (0.322 nm) and indicates that KPCN possesses a denser layered structure. Moreover, the low‐angle reflection peak of PCN at 12.88° was not observed in KPCN, while new peaks appeared at ≈8.05° and 9.89° with the corresponding lattice distance about 1.098 and 0.893 nm, respectively, which could result from the break in the primitive symmetry of PCN caused by the insertion of K ions into the melon chains or layers of PCN (Figure S2, Supporting Information). No peaks were observed that could be assigned to poly(triazine imide) (PTI),^12^ carbon nitride intercalation compound,^13^ and triazine‐based carbon nitride.^14^ Finally, the crystallinities of the samples were evaluated according to a semiquantitative index based on the half‐maximum (FWHM) of the main XRD diffraction peak. KPCN shows a lower FWHM value (0.54) than PCN (0.76), which implies a better crystallinity in KPCN.

**Figure 2:**
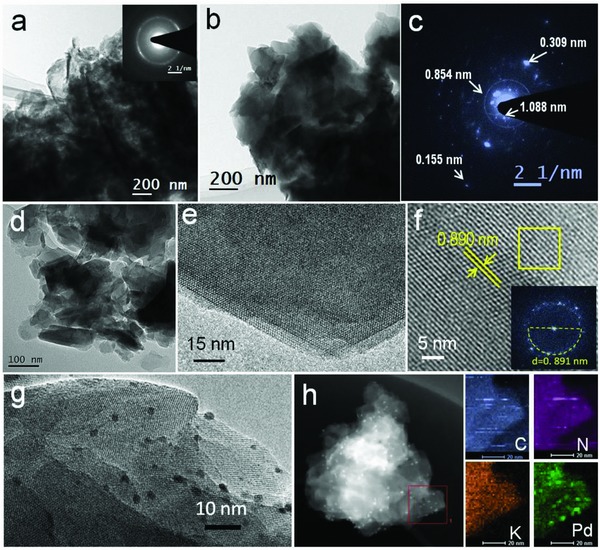
TEM and HRTEM images of a) original PCN, b–f) the synthesized KPCN, and g,h) Pd/KPCN. h) The HAADF‐STEM and element mapping of Pd/KPCN. The inset in (a) and (c) are SAED patterns, the inset in (f) is the FTT pattern from the yellow square of KPCN marked in (f).

**Figure 3:**
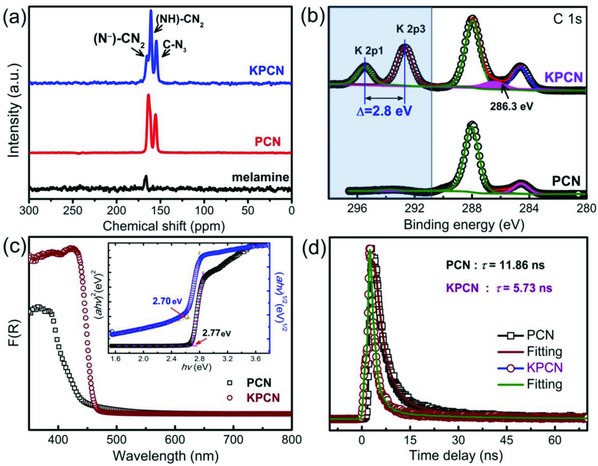
a) Solid‐state ^13^C NMR spectra of melamine, bulk PCN, and the synthesized KPCN. b) The K 2p and C1s XPS spectra of bulk PCN and the synthesized KPCN. c) UV–vis absorption spectra and d) the Kubelka–Munk function plot of KPCN (inset in (c)). d) The time‐resolved fluorescence decay spectra of bulk PCN and KPCN. The lines show the fits according to a biexponential model. The samples were excited with a picosecond pulsed light‐emitting diode (d) at 298 K.

The detailed crystalline structures of the samples were further investigated by high‐resolution transmission electron microscope (HRTEM). As shown in **Figure** **2**, the bulk PCN contained large amorphous aggregates and a layered structure on the micrometer scale (Figure 2a). The KPCN product possessed sheet‐like features stacked in many layers even after ultrasonic treatment (Figure 2b,d; Figure S3a–e, Supporting Information). The selected area electron diffraction (SAED) patterns of KPCN shown in Figure 2c and Figure S3c (Supporting Information) indicated a similar single crystal feature. The bright spots in the centre correspond to two sets of crystal planes with crystal lattice distances of ≈1.088 and 0.854 nm. Another spot, which corresponds to 0.309 nm (cyan circles), can be assigned as the interlayer distance between carbon nitride rings, which is consistent with that determined by XRD (Figure 1b). The HRTEM image provided in Figure 2e clearly exhibit lattice fringes. The crystal interplanar spacing (about 0.890 nm, Figure 2f and the inserted FFT pattern) can be indexed to (110) facet of KPCN at the XRD diffraction angel at 9.89°, which further demonstrates the highly crystalline structure of KPCN. In the composite of Pd/KPCN, the Pd nanoparticles with the mean size of 2 nm (Figure S9 and Table S1, Supporting Information) are homogenously loaded on KPCN (Figure 2g,h; Figure S3g–i, Supporting Information). High angle annular dark field‐TEM (HAADF‐TEM) demonstrated that the K and N are uniformly distributed in KPCN base. ^13^C cross‐polarization (CP)‐magic‐angle spinning (MAS) solid‐state NMR spectrum was next carried out to identify the proposed chemical structure of the tri‐s‐triazine units in the KPCN framework. As shown in **Figure** **3**a, two distinct resonance peaks cantered at 161.2 and 154.4 ppm for KPCN, which are similar to those of melem and melon reported in the literature,^15^, ^16^ could be assigned to the (NH)–CN_2_ structure and C–N_3_ units, indicating the presence of the characteristic poly(tri‐s‐triazine) structures in the KPCN. However, these peaks had a clearly lower shift than those of PCN located at 163.7 and 155.7 ppm, which might be attributed the intercalation of K^+^ and O‐containing functional groups in the C–N framework, as demonstrated by the peak at 172.1 ppm. The composition and chemical states of the prepared KPCN were probed by X‐ray photoelectron spectroscopy (XPS). The full scan reveals that the elements of C, N, O, K, and Br coexisted in both PCN and KPCN (Figure S4a, Supporting Information). The content of graphite‐like sp^2^‐hybridized carbon located at 286.2 eV (Figure 3b), the tertiary nitrogen N–(C)_3_ groups in the heptazine ring and bridging —NH— at ≈399.8 and 400.7 eV (Figure S4b, Supporting Information), respectively,^16^, ^17^ are bigger in KPCN than bulk PCN. K 2p peaks cantered at 292.7 and 295.5 eV were obviously observed (Figure 3b), which are consistent with the reported K 2p binding energies of CH_3_CH_2_OCOOK^18^ and very different from that of metallic K (294.7 eV).^19^ The doublet separation of the K 2p photoelectron line was 2.8 eV, further indicating that K existed in KPCN as K ions coordinated to N atoms located in the intervals of the melon chains in the KPCN (causing a blueshift of ≈0.3 eV in the K 2p binding energy of KBr).^20^

**Figure 4:**
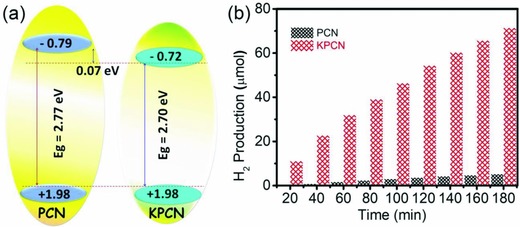
a) Schematic diagram of the determined electronic band alignment. b) Photocatalytic H_2_ evolution over Pd/PCN and Pd/KPCN (1.0 wt% Pd, checked by ICP‐AES) under light irradiation (λ ≥ 280 nm). Reaction conditions: catalyst (20 mg) dispersed in 30 mL methanol/H_2_O solution (volume ratio 1:5) at room temperature with a 500 W Xe lamp as the light source.

Apparently, a highly crystallized KPCN structure would greatly change its chemical and physical properties, for example, light absorption, band structure, charge carrier transfer, etc. First, the diffuse reflectance spectrum was performed (Figure 3c). The results revealed an obvious improvement in the light absorption properties of KPCN (460 nm) over that of powdered PCN (420 nm), reflecting a smaller band gap energy in KPCN (2.70 eV for KPCN vs 2.77 eV for PCN estimated from the Tauc plot, inset of Figure 3c). The VB potential of KPCN was the same as that of PCN (+1.98 eV, **Figure** **4**a and Figure S5, Supporting Information), whereas the CB potential of KPCN was 0.07 eV lower than that of PCN, indicating that salt confined growth changed the reductive activity of excited electrons under light irradiation. Besides, the lower band gap of crystalline KPCN with shortened layer distance than that of the PCN powder, and the electron‐withdrawing group (—C≡N) and inbuilt K ions can facilitate efficient separation of photogenerated charge carriers excited by visible light, as confirmed by the steady‐state and time‐resolved photoluminescence (PL) spectra (Figure S6, Supporting Information). Similar to the results of the steady‐state PL spectra, KPCN exhibited fast fluorescence decay (Figure 3d) with a lifetime of 5.73 ns, which was less than half the lifetime of bulk PCN (11.86 ns). The reduced lifetime could be resulted by the K^+^ insertion or/and the exciton dissociation, which is commonly found in polymeric carbon nitride based systems.^21^ The shorter PL lifetime of KPCN indicates a decrease of exciton population, which further reveals the enhanced exciton dissociation and the reduced electron‐hole recombination on KPCN.^22^ The phenomena of color change (Figure S8, Supporting Information) in hydrogen evolution for water splitting under visible light illumination further confirmed the dissociation of excitons, which improves the separation of electron–hole pairs, and resulting in high photocatalytic activity.

After obtaining the KPCN with good crystalline and electronic structure, improved chemical and physical properties, we subsequently evaluated the H_2_ production activity from water by performing the light‐induced hydrogen evolution assay. The KPCN sample exhibited enhanced photocatalytic activity over that of bulk PCN for hydrogen evolution (Figure 4b). The initial hydrogen evolution rate in the first hour for the optimal KPCN reached 1600 µmol h^−1^ g^−1^ (with the quantum efficiency about 7.3%), which was a factor of 18 times higher than that of the bulk PCN (88 µmol h^−1^ g^−1^). The surface area was not responsible for the enhanced photocatalytic activity. As shown in Figure S7 of the Supporting Information, the highly crystalline KPCN showed an obvious decrease in the surface area (10 m^2^ g^−1^) relative to that of PCN (35 m^2^ g^−1^). Furthermore, the water reduction half‐reaction is related with pH of the reaction solution.^23^ The pH value is about 6.7 in this reaction solution (25 mL H_2_O and 5 mL ethanol) for H_2_ evolution. Accordingly, the conduction band position of KPCN is −0.72 −59 × 10^−3^/6.7 = −0.7288 V. The change of conduction band position of KPCN is just 0.0088 V (−59 × 10^−3^/6.7), which has little effect on the reduction of water. Importantly, a color change was observed in the KPCN catalyst suspension (not in PCN) from yellow to turquoise or blue during irradiation (Figure S8, Supporting Information), which is indicative of a long‐lived excited state.^24^ This state is strongly related to the changed electronic state of KPCN resulting from K^+^ insertion. Based on the aforementioned chemical and physical properties, we can reasonably conclude that the augmented photocatalytic activity resulted from the highly crystalline structure, wide light harvesting capability and appropriate band structure, which promotes the separation of photogenerated carriers and decreases the probability of recombination, in turn increasing the carrier lifetimes.

During water decomposition, the cleavage of an O—H bond in a H_2_O molecule excited by light could produce active H‐species (adsorbed H, H_ad_) that subsequently combine to form molecular H_2._ 25 Likewise, hydrogen isotope species (D‐species, D_2_/D_ad_), could also be produced via a photocatalytic heavy water (^2^H_2_O or D_2_O) splitting process. The above KPCN catalyst exhibited good performance in photocatalytic H_2_ evolution enable us to further achieve our target of utilizing the in situ generated active D‐species from photocatalytic heavy water splitting for the sequential deuteration of alkenes and alkynes under visible light irradiation in ambient conditions. During the deuteration reaction, the photogenerated active D‐species (i.e., D_ad_) could be adsorbed to and stabilized on the surface of metal catalyst, and thus could more easily react with unsaturated organic substrates, such as aromatic olefins, α,β‐unsaturated ketones and alkynes. To develop such a bifunctional catalytic system, Pd nanoparticles (1–3 nm) were uniformly loaded onto KPCN (Figure S9, Supporting Information). The advantages of the KPCN as a photocatalyst and support could be well‐combined in this Pd/KPCN composite bifunctional catalyst. Thus, Pd could not only could act as a cocatalyst for the production of D_2_/D_ad_ but also serve as the main catalyst for sequential catalytic alkenes and alkynes deuteration.

Next, the reaction scope and effectiveness of the above reaction over Pd/KPCN catalyst in the synergistic catalytic system were assessed. As shown in **Table** **1**, deuterium atoms were successfully selectively located into the ethylene or/and ethylene linkage in several typical molecules such as representative aromatic olefins, α,β‐unsaturated ketones, and also alkynes. Representative disubstituted alkenes bearing electron‐donating and electron‐withdrawing substituents such as β‐methylstyrene (**a1**), cinnamonitrile (**a2**), and stilbene (**a3**) were effectively converted to the corresponding deuterated alkanes with high yields (96%, 87%, and 90%, respectively) revealing that the electronic effect had little impact on the efficiency. Representative α,β‐unsaturated ketones such as 3‐methyl‐2‐cyclohexenone (**b1**), acetocinnamone (**b2**), *E*‐chalcone (**b3)**, and 3,4‐dimethoxybenzylideneacetone (**b4**) were also well‐tolerant in the reaction system and afforded the corresponding products in 86–‐89% yields with excellent chemoselectivity.^26^ Besides, some common‐used alkynes such as phenylacetylene (**c1**), 4‐phenyl‐1‐butyne (**c2**), diphenylacetylene (**c3**), and 4‐phenylethynylphthalic anhydride (**c4**) can achieved in high yields (72–92%) with excellent selectivity to the corresponding alkanes. Impressively, when norandrostenedione (**d**), a representative steroidal drug, was also subjected to the reaction, high isolated yields (87%) of products and high deuterated ratio (93%, Figure S10, Supporting Information) at the target molecular sites (C=C linkage) were obtained, approving a promising and novel feasible method for selective incorporation deuteriums in pharmaceutical compounds. The current C–H/C–D exchange strategy is often used to install deuterium in sp or sp^2^ bonds (i.e., aromatic, vinylic, alkynyl) under relatively harsh conditions to achieve high deuteration content. The directing groups are usually required to control the position of deuteration.^27^ Our strategy is able to selectively install deuterium in sp^3^ bonds of fine‐chemicals and pharmaceutical compounds with green and low‐cost D‐source under mild conditions.

**Table 1:**
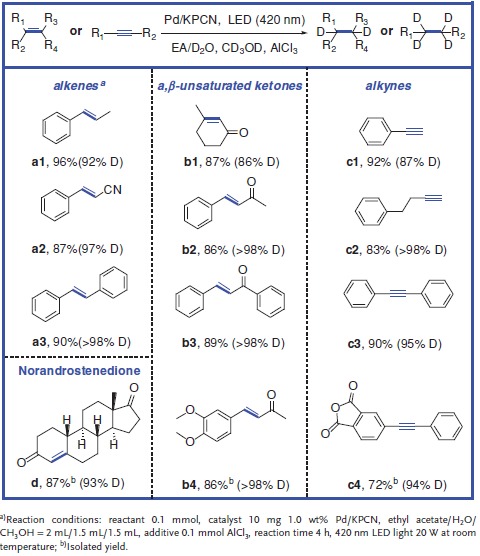
Photocatalytic deuteration of representative alkenes and alkynes

## 3 Conclusions

In summary, we have pioneered a promising integrated photocatalytic system utilizing water splitting technology for selective deuteration of alkenes and alkynes over a crystalline KPCN semiconductor. The present KBr salt‐template‐confined growth process achieved an unprecedented high crystalline KPCN material with remarkably improved light harvesting capabilities and charge carrier separation; thus achieved a significantly enhanced activity over KPCN for visible light photocatalytic H_2_/D_2_ evolution and transfer deuteriation of alkenes/alkynes to furnish high value‐added heavy chemical/pharmaceuticals. We are currently working to better understand the catalytic mechanism and the atomically‐insight into the structure of KPCN and discover new‐generation catalysts that enable us to harness our strategy for widespread artificial photosynthesis of heavy chemicals/drugs.

## 4 Experimental Section

*Preparation of PCN and KPCN*: In a typical synthesis, melamine (3.0 g, Alfa Aesar) was ground with KBr (2.0 g, Alfa Aesar) in 3 mL EtOH and 1 mL glycol in an agate mortar. After drying at 65 °C, the resultant mixture was heated to 550 °C for 3 h at a rate of 3.3 °C min^−1^ in a tube furnace (inner diameter is 5 cm) with open two ends in an air atmosphere. After it was cooled to room temperature, the bright yellow‐green product was washed with boiling deionized water several times and collected by filtration, followed by drying at 60 °C under vacuum. This sample is denoted as KPCN. The samples with different KBr/melamine ration were prepared under the same procedure. For comparison, 3.0 g melamine was directly heated to 550 °C for 3 h without the KBr, which was referred to as PCN.

*Preparation of Pd/PCN, Pd/KPCN Catalysts*: Palladium nanoparticles (1.0 wt%) supported on KPCN (or PCN) were prepared by photodeposition process. In brief, as‐synthesized KPCN (or PCN) (0.3 g) was dispersed in a mix solution with 80 mL deionized water and 20 mL glycol. After untrasonication treatment for 2 h, 28 µL of 1.0 m H_2_PdCl_4_ was added into the mixture, and then the mixture was treated under 500W Xe lamp illumination for 1h to reduce Pd^2+^. The brownish slurry was centrifuged and washed with deionized water at least five times. After dried in an oven at 70 °C overnight under vacuum condition, as‐prepared catalysts denoted as Pd/KPCN (or Pd/PCN) were obtained.

*Characterizations*: X‐ray diffraction (XRD) patterns were performed on Rigaku diffractometer using Cu Kα irradiation at a scan rate of 10 min^−1^ with the target voltage at 30 kV and current 15 mA. TEM images were obtained using a Tecnai F30 (FEI) field emission TEM. The chemical compositions of the samples were analyzed using XPS (Thermo Escalab 250, a monochromatic Al Kα X‐ray source). All binding energies were referenced to the C 1s peak (284.6 eV) arising from adventitious carbon. ICP spectrometry provided an elemental analysis (Ultima 2, Horiba). Brunauer–Emmett–Teller specific surface areas were tested using a Micromeritics ASAP 2020 system. The solid‐state ^13^C solid‐state NMR spectra were recorded on Bruker Avance II instruments using a CP‐MAS sequence mode. The data were referenced to trimethylsilane. The Fourier transform infrared spectra were recorded on a VERTEX70 spectrometer. PL spectra and PL decay profiles of the as‐synthesized powder samples were recorded on an Edinburg Instruments FLS920 spectrofluorometer at room temperature with a 450 W Xenon lamp (excitation: 370 nm) and a 370 nm nanosecond laser, respectively. The element analysis was recorded by vario MICRO. The high‐performance mass spectrometry was conducted by a Q Exactive GC Orbitrap GC‐MS/MS (Thermo Scientific). The quantum efficiency (QE) calculated by the following equation was measured using a 500 W Xe lamp with a band‐pass filter (420 ± 5 nm) at 278 K, and the average intensity of the irradiation wavelength was determined by an optical power meter. QE = (2 × the number of evolved H_2_ molecules/the number of incident photons) × 100%.

*Photocatalytic H_2_ Evolution*: The photocatalytic reactions for water splitting were carried out in a 100 mL Pyrex flask reactor (Suncat instruments Co., Ltd., Beijing, China) via top‐irradiation connected to a closed gas‐evacuation and circulation system. Hydrogen production was performed by dispersing 20 mg of the catalyst powder in an aqueous solution (30 mL) containing 5 mL methanol as sacrificial agent. The reaction solution was evacuated several times to completely remove the air prior to irradiation under a 500 W Xe lamp. The wavelength of the incident light was controlled by applying appropriate long‐pass cutoff filters. The temperature of the reaction solution was maintained at 20 °C using a flow of cooling water during the reaction. The amount of evolved gas was determined online using a gas chromatograph (Fuli 9790II, Zhejiang, China) with TCD detector. The average hydrogen evolution rates were calculated by the equation: H_2_ evolution amount/catalyst amount (20 mg)/reaction time (h).

*Photocatalytic Deuteration of Alkenes and Alkynes*: Typically, 10 mg of Pd/KPCN and 0.1 mmol of substrate and AlCl_3_ were dispersed in a mixture solution with ethyl acetate/D_2_O/CD_3_OD = 2 mL/1.5 mL/1.5 mL, and then sonicated for 1 min. The reaction mixture was then irradiated with an LED lamp (20 W, λ = 420 nm, Suncat instruments Co., Ltd., Beijing, China) for 4 h under Argon at 25 °C by using a flow of cooling water during the reaction. After reaction, the mixture was centrifuged to remove photocatalyst. The supernatant was extracted by adding 5 mL of CH_2_Cl_2_ and the organic phase was analyzed by GC‐MS (Agilent 7890A). The conversions were calculated from standard calibration curves. The isolated yield was calculated by dividing the amount of the obtained desired product. Deuterium incorporation were double checked and calculated by GC‐MS and high performance mass spectrum.

## Conflict of Interest

The authors declare no conflict of interest.

## Supporting information

Supplementary
