# Supplementary material for: Highly Crystalline K‐Intercalated Polymeric Carbon Nitride for Visible‐Light Photocatalytic Alkenes and Alkynes Deuterations
Source: Adv Sci (Weinh). 2018 Nov 8;6(1):1801403. doi: 10.1002/advs.201801403 (PMC6325627; doi:10.1002/advs.201801403)
Supplement: Supplementary file 1 — Supplementary [file ADVS-6-1801403-s001.pdf]

Copyright WILEY-VCH Verlag GmbH & Co. KGaA, 69469 Weinheim, Germany, 2018.

ADVANCED SCIENCE
Open Access

Supporting Information

for *Adv. Sci.,* DOI: 10.1002/advs.201801403

Highly Crystalline K-Intercalated Polymeric Carbon Nitride for Visible-Light Photocatalytic Alkenes and Alkynes Deuterations

*Chuntian Qiu, Yangsen Xu, Xin Fan, Dong Xu, Rika Tandiana, Xiang Ling, Yanan Jiang, Cuibo Liu, Lei Yu, Wei Chen, and Chenliang Su**

Copyright WILEY-VCH Verlag GmbH & Co. KGaA, 69469 Weinheim, Germany, 2016.

Supporting Information

Highly Crystalline K-Intercalated Polymeric Carbon Nitride for Visible-light Photocatalytic Alkenes and Alkynes Deuterations

*Chuntian Qiu,※ Yangsen Xu, ※ Xin Fan, Dong Xu, Rika Tandiana, Xiang Ling, Yanan Jiang, Cuibo Liu, Lei Yu, Wei Chen, Chenliang Su**

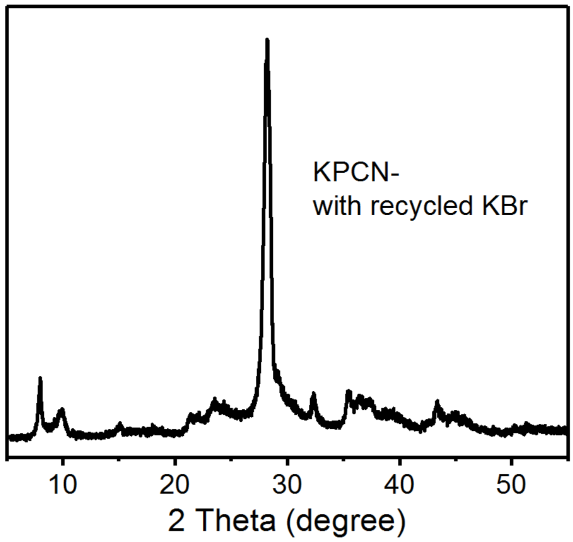

Figure S1 the XRD pattern of KPCN prepared with the recycled KBr. It shows that the crystallinity and the structure did not changed and has the same result as that with fresh KBr in the precursors.

In this work, we used thermal condensation of melamine with KBr which is different from the reported molten salt process that involved two or three salt forming reaction solution. KBr here keeps in solid state and acts as a solid temple to direct the growth of crystalline KPCN. One advantage of using KBr as a solid template is that it can be dissolved in boiling water and very easily removed, leaving behind pure, light yellow-green KPCN after repeated rinsing and filtering. The recrystallized KBr salt could be reused to prepare KPCN when combined with melamine indicating that KBr crystals provide an economical and universal strategy for the growth of KPCN.

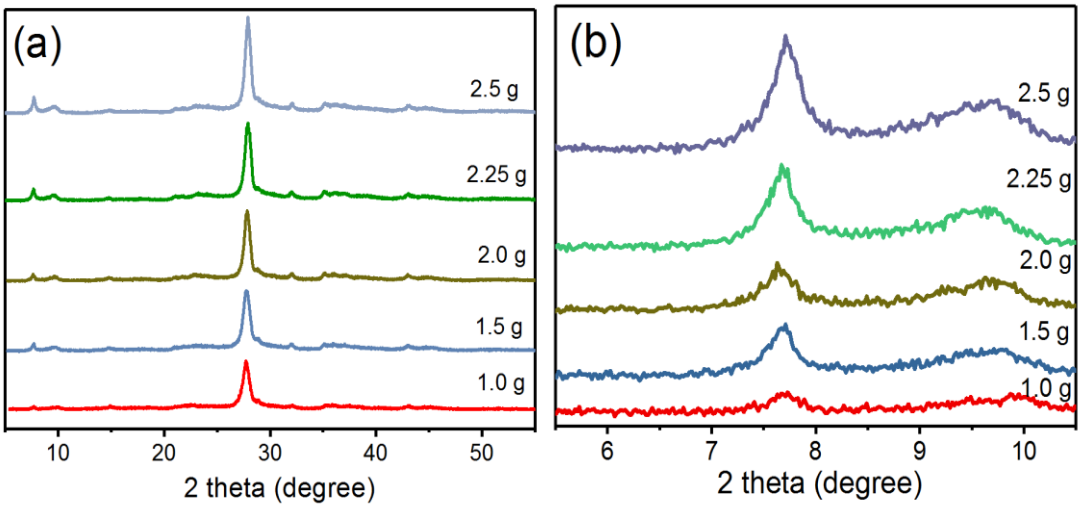

Figure S2 the XRD patterns of the obtained KPCN prepared with different loading of KBr in the precursors.

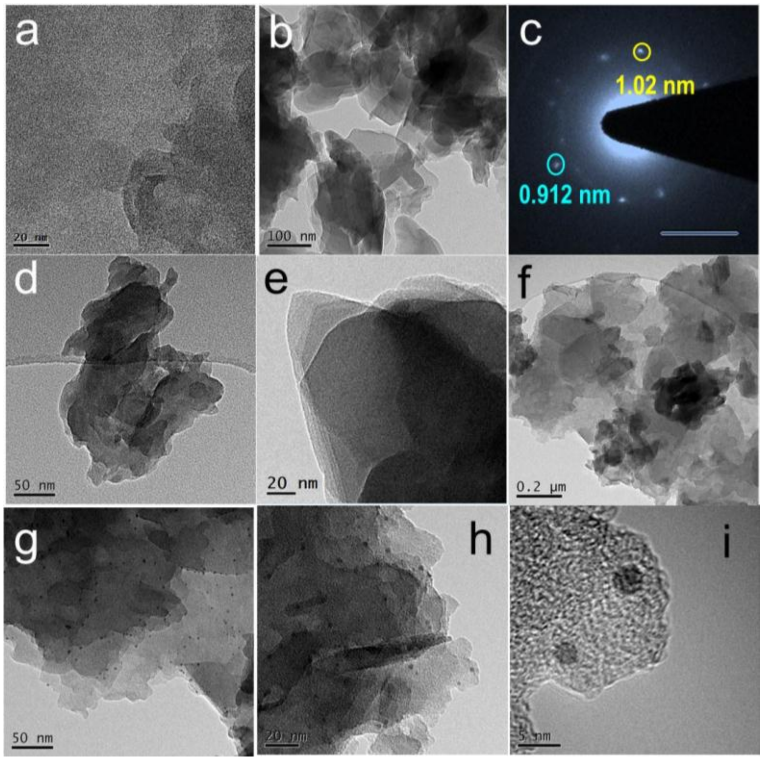

Figure S3 HRTEM of the bulk PCN (a), the synthesized KPCN (b-e) and the Pd/KPCN (f-i).

Figure S3a shows an amorphous state of bulk PCN under the pyrolysis process from melamine. The TEM imagines of KPCN in Figure S3b-e clearly show a layered feature consist of many layer of carbon nitride and crystalline structure. After ultrasonication treatment for 2 h, the layered structure maintained (Fig.S3f) and the loaded Pd nanoparticles are uniform anchored on the KPCN base (Fig.S3g-i).

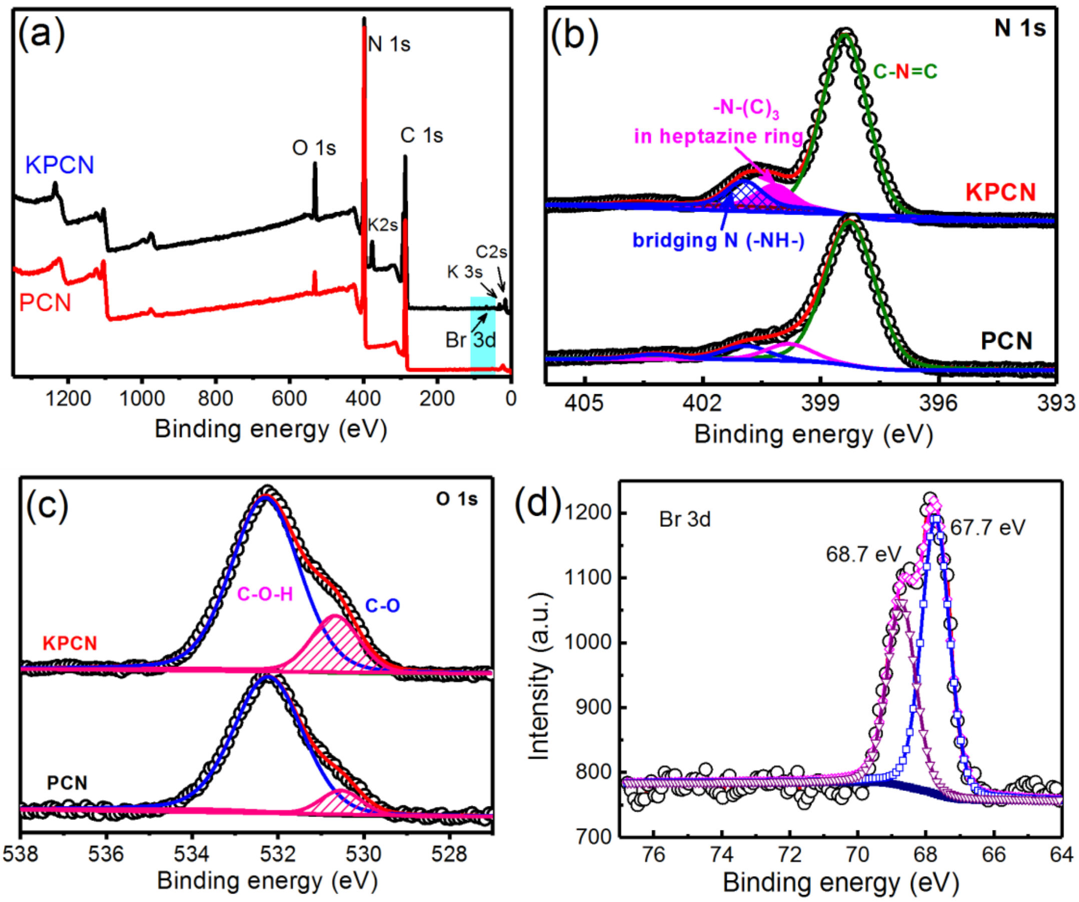

Figure S4 XPS patterns of overview PCN and KPCN (a) and the XPS C 1s (b), O1s (c) and Br 3d (d) in KPCN. The chemical state of Br in KPCN can be indexed to the ionic state like in the KBr.

The full scan (Figure S4a) reveals that the elements of C, N and O coexisted in both PCN and KPCN, while K was present only in the latter. The high-resolution N 1s spectra (Figure S4b) could be separated into three peaks. The strongest peak (398.3 eV) was due to the sp^2-hybridized nitrogen in the tri-s-triazine ring (C-N=C). A blueshift in this peak relative to that of PCN of approximately 0.2 eV may be ascribed to the coordination of K^+ to N.

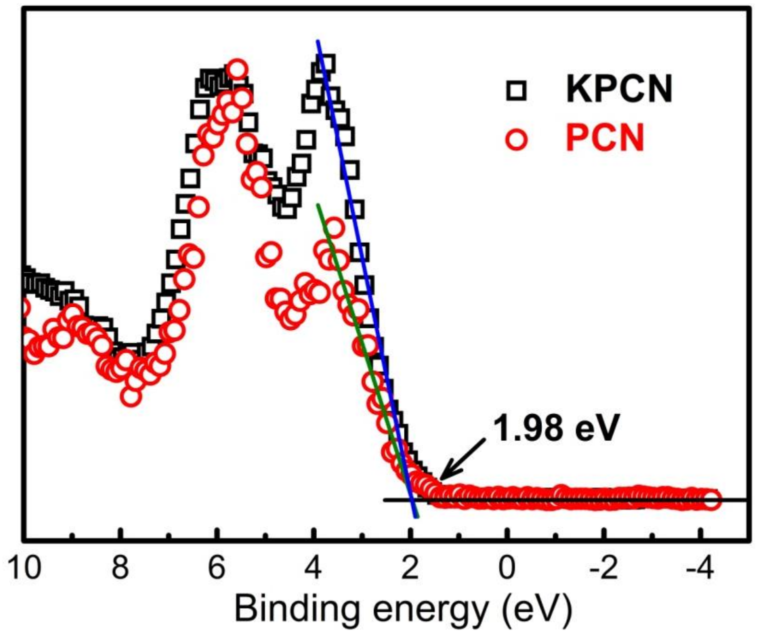

Figure S5 XPS valence band spectra of bulk PCN and KPCN. It can be found that the as-obtained PCN and KPCN have the same VB potential (1.98 eV).

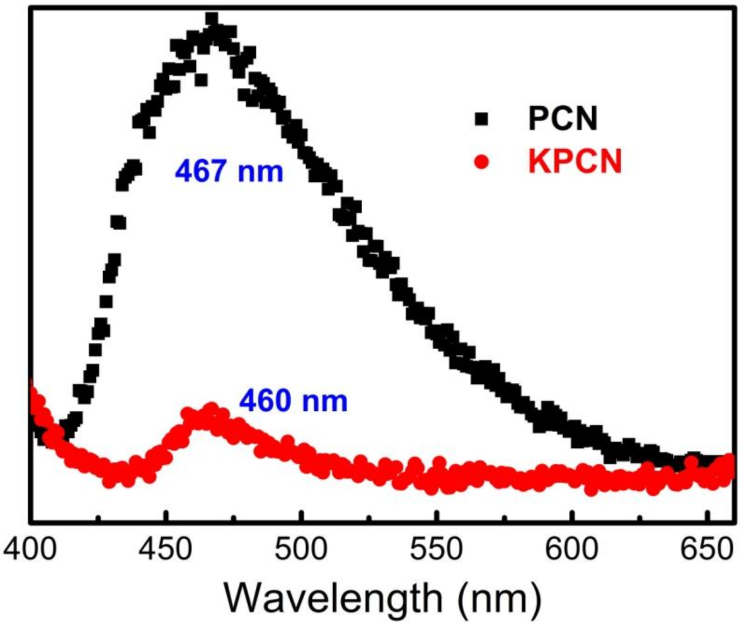

Figure S6 PL spectra of bulk PCN and KPCN.

The bulk PCN displayed a typical absorption pattern of an organic semiconductor, with strong band gap absorption at approximately 467 nm. The obtained KPCN shown a PL emission peak centred approximately 460 nm that was weaker than the emission peak of bulk PCN, suggesting low radiative recombination of the photoexcited electrons and holes.

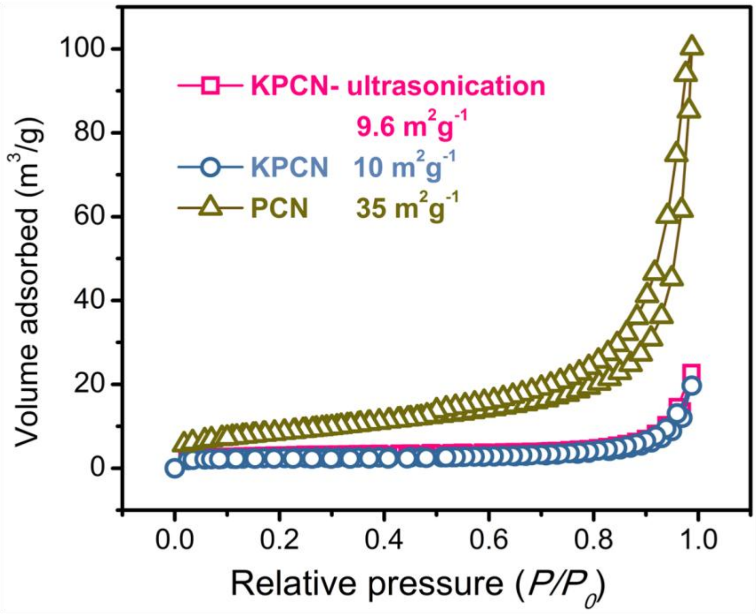

Figure S7 N_2 adsorption-desorption isotherms of the bulk PCN and synthesized KPCN.

An obvious decreased BET surface area can be found from PCN (35 m^2g^{-1}) to crystalline KPCN (10 m^2g^{-1}). Additionally, the ultrasonic treatment did not enhance the BET surface area and shows almost the same value as the KPCN.

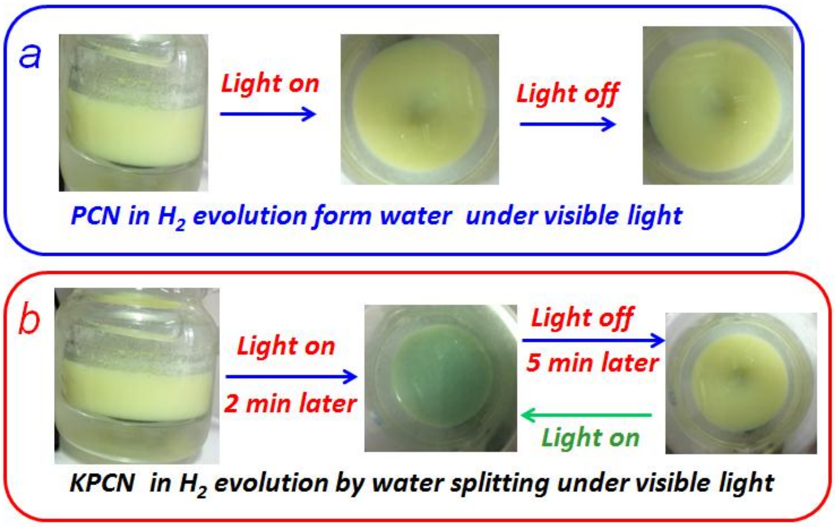

Figure S8 The color change of PCN and KPCN dispersed in 10vol% TEOA solution, with 1.0wt% Pt as cocatalyst under visible-light irradiation (300 W Xe lamp, 420 nm $\leq\lambda\leq$780nm) at 5 ^{o}C.

During the water splitting, the color of PCN aqueous dispersion keep yellow, but the KPCN suspension becomes turquoise or blue from yellow during irradiation, which indicating of a long-lived excited electron state in KPCN thus boost the photocatalystic activity.

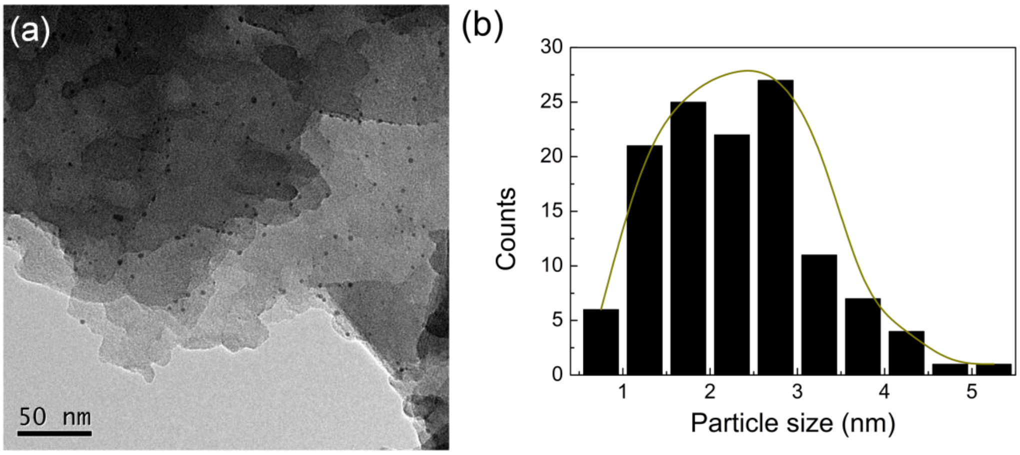

Figure S9 (a) HRTEM image of Pd/KPCN photocatalyst and (b) sizes distribution of Pd nanoparticles.

Table S1 Statistics description of Pd nanoparticle

Total number	Mean (nm)	Standard Deviation	Sum. (nm)	Minimum (nm)	Median (nm)	Maxium (nm)
125	2.29	0.91	286.63	0.63	2.24	5.01

Results clearly exhibits lattice fringes indicating a well defined crystalline structure of KPCN, and the average particle size of Pd on KPCN is approximate to 2 nm.

Table S2 optimization of solvents

Solvent	Ethyl acetate	CH_3CN	Acetone	DMF	Diethyl ether	DMSO
Yield (%)	51	32	42	15	20	9

Reaction conditions: reactant 0.1 mmol of α-methylstyrene, catalyst 15 mg Pd/KPCN (Pd 1 wt%), Solvent/H_2O/CH_3OH=2mL/1.5mL/1.5mL, additive 0.1mmol H_2SO4, reaction time 1h, 420nm LED light 20W at 25°C.

Deuterated product

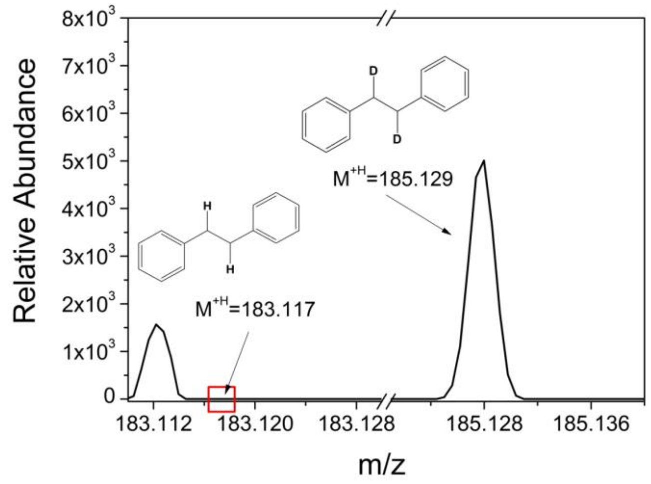

Deuterated product

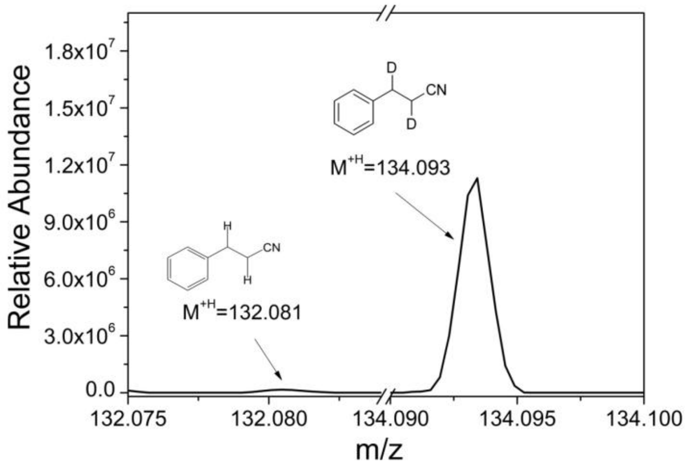

Deuterated product

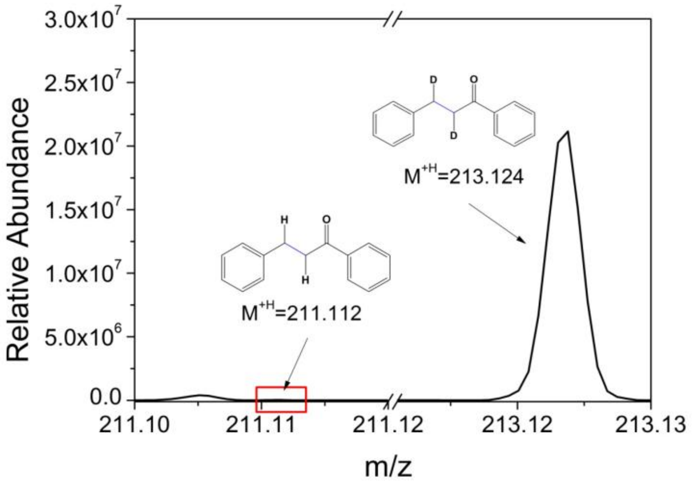

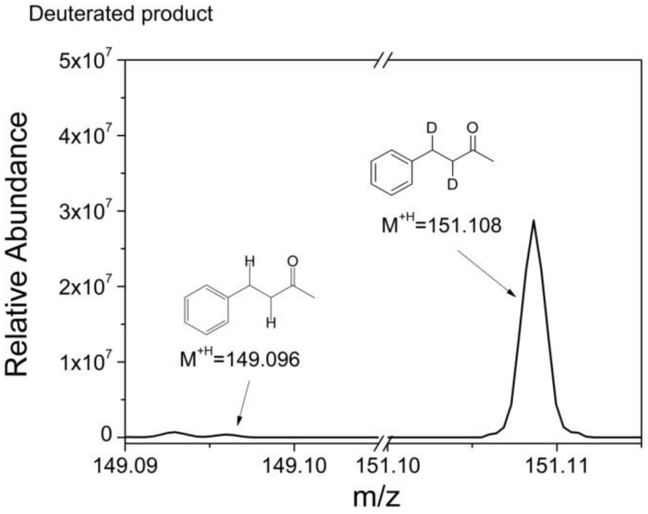
Deuterated product
5x10^7
4x10^7
3x10^7
2x10^7
1x10^7
0
Relative Abundance
M^+H=151.108
M^+H=149.096
149.09
149.10
151.10
151.11
m/z

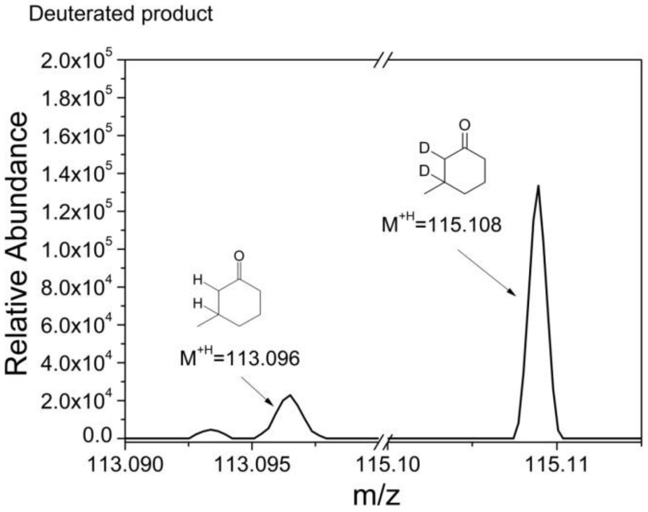
Deuterated product
2.0x10^5
1.8x10^5
1.6x10^5
1.4x10^5
1.2x10^5
1.0x10^5
8.0x10^4
6.0x10^4
4.0x10^4
2.0x10^4
0.0
Relative Abundance
M^+H=115.108
M^+H=113.096
113.090
113.095
115.10
115.11
m/z

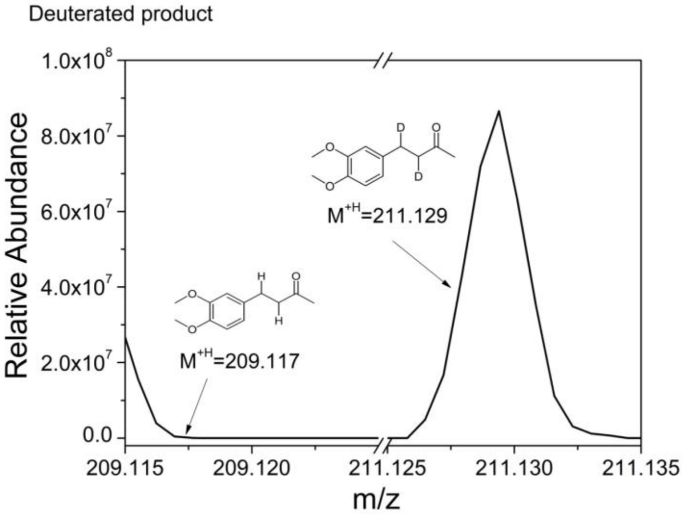
Deuterated product
1.0x10^8
8.0x10^7
6.0x10^7
4.0x10^7
2.0x10^7
0.0
Relative Abundance
M^+H=211.129
M^+H=209.117
209.115
209.120
211.125
211.130
211.135
m/z

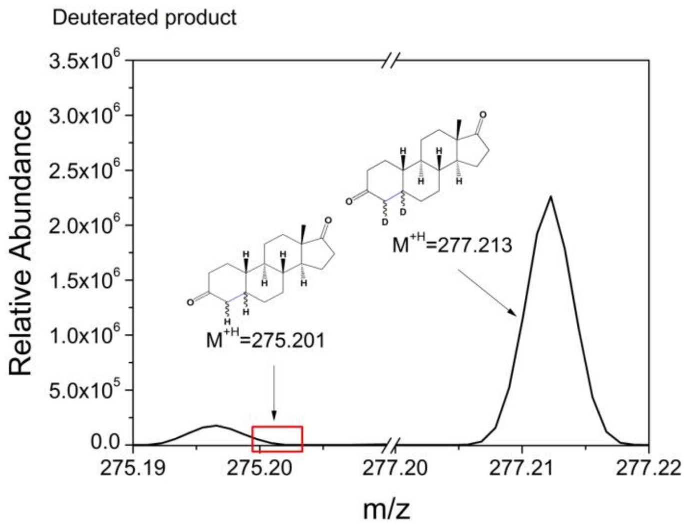

Figure S10 high-performance mass spectrometry of deuterated compounds.

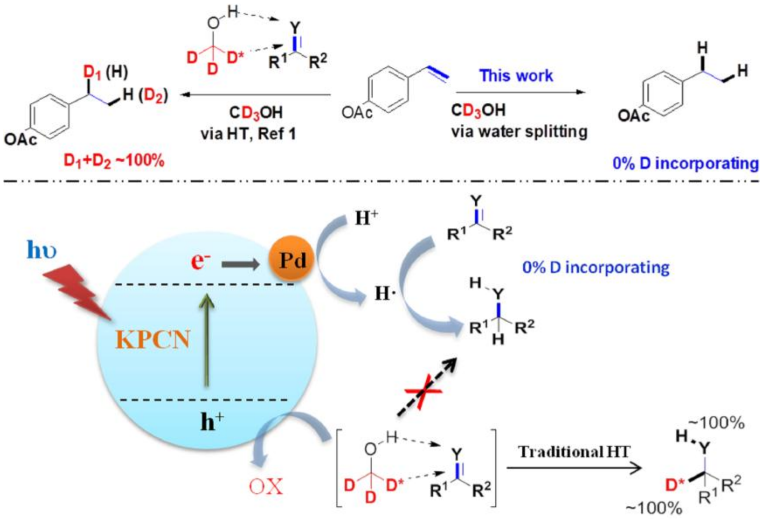

Scheme S1 Mechanistic comparison of hydrogenation via hydrogen transfer and water splitting pathway

To identify the catalytic mechanism, isotopic labeling experiments were conducted, and a putative catalytic pathway for the deuteration/hydrogenation of alkenes is proposed in Scheme S1. In a traditional deuterium/hydrogen transfer mechanism, both the β-C-D and O-H in alcohol are fully transferred to become the β-C-D and Y-H in product.[1] On the contrary, if the deuteration/hydrogenation of alkenes is proceeded by utilizing the D-source/H-source generated *in-situ* from water splitting as we proposed, the proton in aqueous solution will play a critical role and therefore no deuterated labelling product should be obtained. Experiment using CD_3OH/H_2O as the D-source/H-source was carried out accordingly. As expected, no deuterated labelling product can be observed from NMR as well as high-performance mass spectrometry (Figure S11-S12). This result is well-consistent with our proposed mechanism, in which D_{ad}/H_{ad} species is firstly generated and then attached on the surface of Pd nanoparticles for cascade deuteration/hydrogenation of alkenes.

[1] a) J. S. M. Samec, J. E. Bàckvall, P. G. Andersson, P. Brandt, *Chem. Soc. Rev.* **2005**, *35*, 237; b) E.A. Serafino Gladiali, *Chem. Soc. Rev.* **2006**, *35*, 226. c) G.M. Grazia Zassinovich, *Chem. Rev.* **1992**, *92*, 1051. d) X. Ma, C. Su, Q. Xu, *Top. Curr. Chem.* **2016**, *374*, 27.

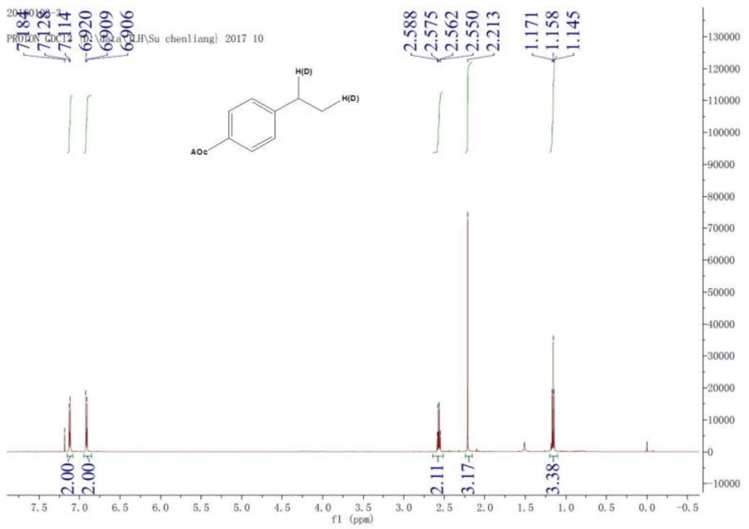

Figure S11 NMR spectra of hydrogenated 4-acetoxystyrene using H_2O/CD_3OH

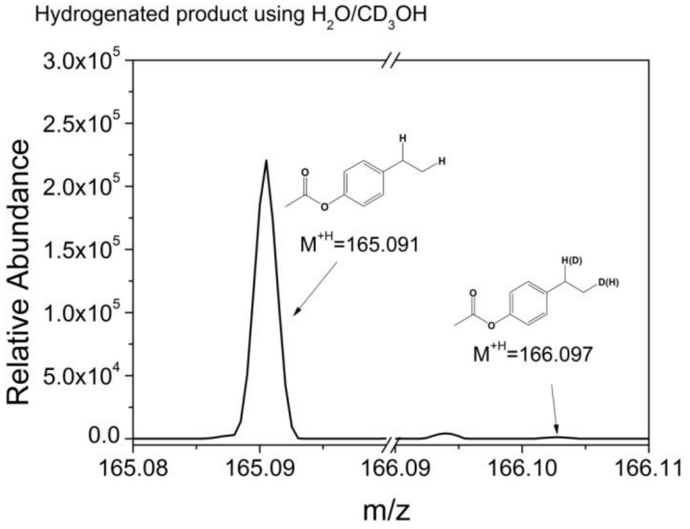

Figure S12 high-performance mass spectrometry result of hydrogenated 4-acetoxystyrene using H_2O/CD_3OH

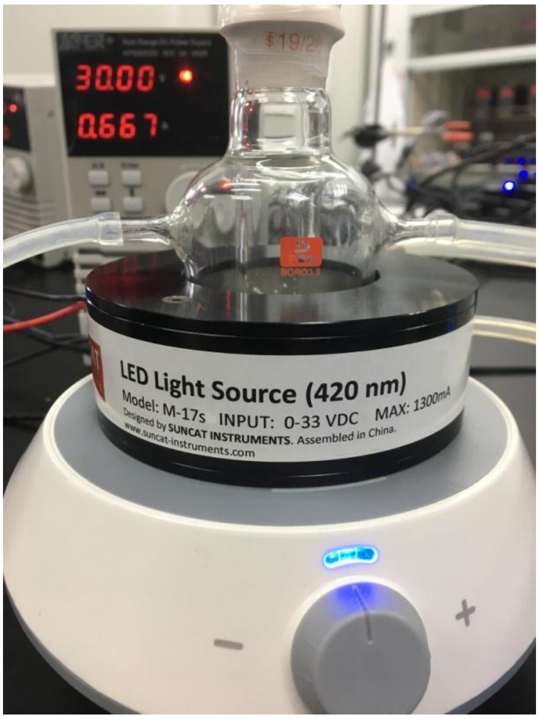

Figure S13 photograph of the reaction setup.
